# Primary Congenital and Childhood Glaucoma—A Complex Clinical Picture and Surgical Management

**DOI:** 10.3390/diagnostics15030308

**Published:** 2025-01-28

**Authors:** Valeria Coviltir, Maria Cristina Marinescu, Bianca Maria Urse, Miruna Gabriela Burcel

**Affiliations:** 1 Ophthalmology Discipline, Carol Davila University of Medicine and Pharmacy, 8 Eroii Sanitari Blvd, 050474 Bucharest, Romania; 2 Clinical Hospital for Ophthalmological Emergencies, 1 Lahovari Place, 010464 Bucharest, Romania; 3 Medical Physiology Discipline, Carol Davila University of Medicine and Pharmacy, 8 Eroii Sanitari Blvd, 050474 Bucharest, Romania; 4 Faculty of Medicine, Transilvania University of Brasov, 56 Nicolae Bălcescu Street, 500019 Brasov, Romania; 5 Brasov County Emergency Clinical Hospital, 25 București Avenue, 500326 Brasov, Romania

**Keywords:** congenital glaucoma, buphthalmos, trabeculectomy, goniotomy, glaucoma drainage devices

## Abstract

Childhood glaucoma encompasses a group of rare but severe ocular disorders characterized by increased intraocular pressure (IOP), posing significant risks to vision and quality of life. Primary congenital glaucoma has a prevalence of one in 10,000–68,000 people in Western countries. More worryingly, it is responsible for 5–18% of all childhood blindness cases. According to the Childhood Glaucoma Research Network (CGRN), this spectrum of disease is classified into primary glaucoma (primary congenital glaucoma and juvenile open-angle glaucoma) and secondary glaucomas (associated with non-acquired ocular anomalies, non-acquired systemic disease, acquired conditions, and glaucoma after cataract surgery). They present very specific ocular characteristics, such as buphthalmos or progressive myopic shift, corneal modifications such as Haab striae, corneal edema or increased corneal diameter, and also glaucoma findings including high intraocular pressure, specific visual fields abnormalities, and optic nerve damage such as increased cup-disc ratio, cup-disc ratio asymmetry of at least 0.2 and focal rim thinning. Surgical intervention remains the cornerstone of treatment, and initial surgical options include angle surgeries such as goniotomy and trabeculotomy, aimed at improving aqueous outflow. For refractory cases, trabeculectomy and glaucoma drainage devices (GDDs) serve as second-line therapies. Advanced cases may require cyclodestructive procedures, including transscleral cyclophotocoagulation, reserved for eyes with limited visual potential. All in all, with appropriate management, the prognosis of PCG may be quite favorable: stationary disease has been reported in 90.3% of cases after one year, with a median visual acuity in the better eye of 20/30. Immediate recognition of the specific signs and symptoms by caregivers, primary care providers, and ophthalmologists, followed by prompt diagnosis, comprehensive surgical planning, and involving the caregivers in the follow-up schedule remain critical for optimizing outcomes in childhood glaucoma management.

## 1. Introduction

Glaucoma is a widely spread disease worldwide, with characteristic damage in the retinal ganglion cell layer and specific visual field defects, and is a leading etiology of blindness [1]. In particular, childhood glaucoma represents a group of disorders of the eye characterized by increased intraocular pressure (IOP), with a severe impact on ocular structures [2].

Childhood glaucomas are rare, but they pose a significant risk to the child’s visual ability and their general wellbeing. Vision-related quality of life (QoL) encompasses the ability to perform vision-dependent tasks such as reading, writing, and general schoolwork but also the impact of vision on emotional and social wellbeing. It has been shown that in childhood glaucoma, QoL is decreased, and a better QoL is associated with better visual acuity [3].

Furthermore, childhood glaucoma presents a series of diagnostic challenges that may impede the timely identification and treatment of cases. Reasons for delayed presentation include low socioeconomic conditions and difficult access to healthcare centers; a study reported that, on average, cases that were recognized by caretakers from the first weeks of life presented to tertiary care centers with an average delay of 3 months [4]. Diagnosis is further complicated by the multiple diagnosis tools, poor collaboration of the child, and the need for general anesthesia in order to properly examine the potential patient [5]. Modern tools may prove valuable in aiding timely diagnosis, and a deep-learning model that analyses primary gaze photographs of children has reached a sensitivity of 0.85 and specificity of 0.94 [5]. Moreover, while the clinical picture may seem obvious to childhood glaucoma, several clinical pictures can confuse the clinician. In a young child, cooperation is difficult to establish; therefore, the intraocular pressure may read falsely high [6]. The appearance of an enlarged eyeball can be a feature of other ocular pathologies (megalocornea, keratoglobus, high myopia, or inflammation sequelae), and corneal findings may be also due to birth trauma, corneal dystrophies, lysosomal storage disease, Peters anomaly or sclerocornea, and optic nerve findings may be due to physiological cupping, papillorenal syndrome, or optic nerve hypoplasia following cerebral intrauterine hypoxic-ischemic injury [6].

The objective of this work is to provide a review of the current classification of childhood glaucomas, accompanied by an efficient overview of the clinical picture and of the proper medical and surgical management.

## 2. Classification and Epidemiology of Congenital Glaucoma

Congenital glaucoma is a complex disease, with variable ocular, systemic manifestations, and age of onset. Classically, adult glaucomas are further divided into angle closure and open angle glaucomas (either primary or secondary) [7], and while childhood glaucoma can also occur through these two mechanisms, the Childhood Glaucoma Research Network (CGRN) has unified the varying classification systems into primary and secondary childhood glaucomas. Primary glaucoma includes primary congenital glaucoma (PCG) and juvenile open-angle glaucoma (JOAG) [8]. Secondary glaucoma is classified as glaucoma associated with non-acquired ocular anomalies (for example, Axenfeld–Rieger Syndrome), glaucoma associated with non-acquired systemic disease (such as connective tissue disorders), glaucoma associated with acquired conditions, and glaucoma after cataract surgery [8,9].

**Figure 1:**
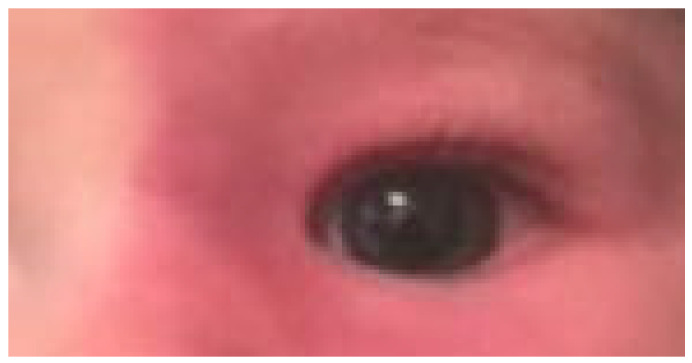
Buphthalmic aspect of an eye in a patient with secondary childhood glaucoma associated with systemic diseases or syndromes, in this case, Sturge–Weber Syndrome (personal archive of author V.C.).

PCG is considered the most common glaucoma in children, not associated with other syndromic manifestations. Usually, it is inherited in an autosomal recessive manner, with a positive family history in cases of consanguinity [10,11]. JOAG is usually diagnosed after the age of 4 years old, with no other ocular and systemic abnormalities and with a normal aspect of gonioscopy [10]. In the case of non-acquired ocular anomalies, they may often associate with glaucoma and are usually managed medically, except when the glaucoma debut is very early, and the case needs to be managed surgically [10]. Similarly, non-acquired systemic diseases or syndromes may be associated with glaucoma and need consistent ophthalmological follow-up throughout life in order to detect increased IOP (for example, Sturge–Weber Syndrome, or neurofibromatosis, homocystinuria, or Marfan syndrome)—see Figure 1 [10]. Lastly, glaucoma may develop secondary to cataract surgery or after other ocular anomalies: uveitis, ocular trauma, corticosteroid treatment, retinopathy of prematurity, and intraocular tumors [10].

It is estimated that primary congenital glaucoma affects one patient in 10,000–68,000 people in Western countries. More worryingly, it is responsible for 5% of all childhood blindness cases [12], with certain research citing values of even 18% of childhood blindness cases worldwide [13]. Among children, the prevalence is higher: 2.7% in Europe and up to 8.1% in South Asia and India [14].

The genetic etiology of childhood glaucoma is complex, with several genes responsible for variable expression and overlapping clinical pictures. Most commonly, genetic testing reveals mutations in CYP1B1, LTBP2, TEK (in PCG) [10], MYOC, TBK1, OPTN (in JOAG) [10] and a variety of mutations in glaucoma associated with non-acquired ocular anomalies, FOXC1, PITX2 (in Axenfeld–Rieger Anomaly And Syndrome) [10], PAX6 (in aniridia) [10], and LTBP2 and CPAMD8 [8,10].

The etiopathogenesis of PCG essentially implies a maldevelopment of AH outflow pathways, particularly in the trabecular meshwork [15]. Histopathological analyses of the trabecular meshwork in PCG show several abnormalities, such as membranes overlaying the angle, high iris insertion, decreased intertrabecular spaces with large trabeculae, and abnormalities of the Schlemm’s canal [16]. Anterior segment optical coherence tomography and ultrasound biomicroscopy are also valuable in this aspect, reporting abnormalities such as hyperreflective membranes over the angle, an absence of Schlemm’s canal, and an underdeveloped ciliary body [16]. While more research is needed to truly decipher the pathogenesis of PCG, it is thought that the iridocorneal angle aspect is due to the abnormal maturation of tissues derived from the cranial neural crest [10].

## 3. Clinical and Paraclinical Diagnosis of Congenital Glaucoma

Symptoms that should alert the children’s caregivers and primary care physicians are the following: photophobia, excessive tearing, and blepharospasm. A fussy crying infant, with no other discernible cause, may also raise the suspicion of congenital glaucoma [17]. Furthermore, the enlargement of the eye (called buphthalmos) should indicate a full ophthalmic assessment of the child [17].

**Figure 2:**
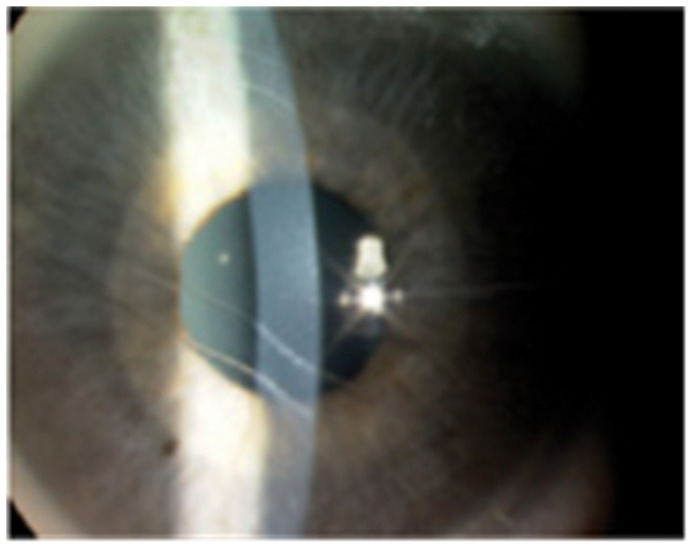
Slit-lamp photograph of Haab striae (Personal archive of author V.C.).

The Childhood Glaucoma Research Network (CGRN) has defined childhood glaucoma based on two criteria [9]. The diagnosis should be made during childhood (under 18 or 16 years old, based on national criteria) and at least two of the following ocular characteristics should be present: intraocular pressure over 21 mmHg; field defect specific to the glaucomatous optic neuropathy; progressive myopia or myopic shift, surpassing the normal growth rate for the age (objectified using the axial length); corneal findings (Haab striae—Figure 2, corneal edema, increased corneal diameter—over 10.5 mm at birth and over 12 mm under the age of 1 year old [17]); and optic nerve findings (progressive increase in cup-disc ratio, cup-disc ratio asymmetry of at least 0.2, focal rim thinning) [9,18]. The increased cup-disc ratio may improve as the IOP is therapeutically decreased; however, this is not an ideal prognostic marker, as glaucoma progression may still occur after cupping reversal [19].

The increase in corneal diameter appears if the glaucomatous insult occurs before the age of 3 years old, and measuring the diameter periodically may assist in establishing the diagnosis and monitoring progression and response to treatment [10]. The investigation of the axial length (AL) and the refractive status of the patient may act as a valuable proxy for diagnosis and follow-up of congenital glaucoma. High AL (>20 mm at birth or >22 mm after the age of 1 year) [17], outside the normal range for age, is a strong indicator of glaucoma. In diagnosed glaucoma, a chronic increase in AL or an increase in the degree of myopia may indicate that the IOP is not under control [10,20].

A crucial investigation in a congenital glaucoma suspect is the gonioscopy (a method of viewing the elements of the anterior chamber angle) [17]. In congenital glaucoma, it may reveal poor differentiation of normal structures, an anterior iris insertion, or prominent iris processes [17]. Other modifications visualized using a gonioscopy lens or mirror are either vascular loops in the iridotrabecular angle or a fluffy fine tissue overlying the peripheral iris [21].

As the visual field defect represents a crucial diagnostic element, it is important to obtain such data in childhood glaucoma patients. However, performing this investigation is difficult for children, as they can become fatigued easily and their shorter attention span may lead to unreliable visual fields. It has been reported that, under careful guidance, reliable visual fields can be obtained in children as young as 5 years old [22]. A large study involving PCG shows that 41% of cases of treated, controlled PCG present definitive glaucomatous defects, the most common being arcuate scotoma (22%). Moreover, children who, at presentation, had an IOP of over 30 mmHg or were diagnosed under the age of 1 month old (compared to those diagnosed over the age of 1 year old) had significantly more affected visual fields [22].

Additional paraclinical investigations in a congenital glaucoma patient include ocular echography (mainly useful to determine the axial length and follow the elongation of the globe) and investigations for the anterior segment morphology (ultrasound biomicroscopy and anterior segment optical coherence tomography) [19]. The latter two are useful in further clarifying the morphology of the iridotrabecular angle and the aspect of trabeculodysgenesis and may aid in surgical planning [19].

Ultrasound biomicroscopy may reveal details on the aspect of the iridotrabecular angle. A large review has synthesized the modification in PCG: an open angle with a thinner iris, the Schlemm’s canal is narrower or even absent, the zonules are longer, and frequently the iris is abnormally inserted or the trabecular meshwork is covered by excessive tissue [23].

Optical coherence tomography (OCT) is also useful in evaluating glaucomatous damage at the level of the optic nerve. The evaluation of certain structures in the retina (ganglion cell layer, inner plexiform layer, and retinal nerve fiber layer) has been identified as an accurate disease marker in childhood glaucoma; however, its usage is limited by the cooperation required of the child and by the lack of normative data in this age group [19].

## 4. Treatment of Congenital Glaucoma

In congenital glaucoma, most often the first line of treatment includes surgery [11,12]. However, following a partially successful surgery, topical treatment plays a crucial role: the proportion of cases with controlled IOP increases from 60 to 94% in primary congenital glaucoma and from 28 to 86% in secondary glaucoma [24]. Moreover, medical treatment may be the only option in patients with a high anesthetic risk or when it is needed to postpone the surgery, in order to have a reduction in IOP, in corneal edema, and, thus, better surgical visualization [14,18].

It is reported that, in children, the efficacy of decreasing the IOP is 20–23% for the therapeutic class of carbonic anhydrase inhibitors, 9–36% for beta-blockers, and 26–27% for prostaglandin analogues, however, with a response rate between 38 and 83% [14]. In terms of surgery, the main types recognized by the World Glaucoma Association 9th Consensus Meeting on Childhood Glaucoma are angle surgeries (goniotomy and trabeculotomy)—the first line of treatment in primary congenital glaucoma, trabeculectomy (usually combined with antifibrotic agents), the implantation of glaucoma drainage devices (GDD), and laser cyclophotocoagulation [10], see Table 1.

Besides the direct treatment of the increased IOP, childhood glaucoma patients often require aggressive visual rehabilitation measures, such as the treatment of amblyopia and the treatment of refractive errors often associated with glaucoma (myopia and astigmatism) [27] refractive errors, which may, themselves, involve severe ocular complications [28].

### 4.1. Goniotomy

Goniotomy is a widely performed surgery in childhood glaucoma cases. It is recommended mainly in PCG, but also in certain cases with a later onset, in certain etiologies of Glaucoma associated with non-acquired ocular anomalies (e.g., aniridia, iris hypoplasia), certain etiologies of Glaucoma associated with non-acquired systemic disease (i.e., Sturge–Weber Syndrome), in Glaucoma associated with acquired condition (uveitic or steroid-induced glaucoma), and also in Glaucoma following cataract surgery [18]. Good angle visualization is essential: corneal edema thus represents a relative contraindication [18,25]. The role of this surgery is to increase aqueous outflow with a lower resistance through the trabecular meshwork to the Schlemm’s canal [18,25].

The preoperative topical treatment involved Apraclonidine 0.5%, in order to decrease blood reflux in episcleral veins throughout the surgery when the eye is expected to be hypotonous, and Pilocarpine 1%, in order to constrict the pupil, protect the lens, and expose the angle [18]. In the case of persistent epithelial corneal edema, an epithelial debridement is performed on a small area of the cornea in order to allow visualization [18]. A direct gonioscopy lens is applied to the cornea, a 23–25G needle or a goniotomy knife is used to create a tunnel through the temporal portion of the cornea, and the depth of the anterior chamber is managed using either viscoelastic material or a maintainer irrigating with balanced salt solution (BSS). The instrument is advanced toward the nasal part of the angle and a cut is created circumferentially between the iris root and the Schwalbe. A bright white line (the goniotomy cleft) should appear [18,25]. The instrument is withdrawn from the eye and the anterior chamber is reformed, in order to regain a normal IOP. The incision site is secured either by hydrating the corneal stroma around the incision (self-sealing) or by performing a 10-0 vicryl suture [18,25].

Reported goniotomy success rates vary between 30% and 90%, with a lower level of success in the case of very early onset [25]. Common complications include hyphema (bleeding in the anterior chamber), and more rarely, patients may develop peripheral anterior synechiae (adhesions between the iris and trabecular meshwork), iridocyclodialysis (separation of the iris and ciliary muscle from the scleral spur), lens injury, or retinal detachment [25].

### 4.2. Trabeculotomy and Trabeculectomy

The procedure of trabeculotomy has been developed as an alternative to goniotomy, in cases in which corneal edema does not allow for good angle visualization [27]. This technique uses an ab externo approach in order to cannulate the Schlemm’s canal and break its wall [27]. The procedure has been proven to lead to lower IOP if treating a higher circumference of the iridotrabecular angle [27]. Several variations to perform the trabeculotomy have been developed, such as using a classical trabeculotome, a 6-0 prolene suture filament, or an illuminated microcatheter [27]. The latter two variations can also be performed ab interno [18].

Moreover, trabeculotomy may be combined with the classical glaucoma procedure trabeculectomy, or with other procedures, such as a deep sclerectomy or viscodilation of Schlemm’s canal [18].

Similar to goniotomy, trabeculotomy is used primarily in PCG but has also been proven to be efficient in JOAG, glaucoma following cataract extraction, and secondary glaucoma due to steroids, uveitis, and in glaucoma associated with Sturge–Weber Syndrome [18]. Also, the internal approach of the surgery is contraindicated in cases of poor visualization (corneal edema, hyphema) and in closed-angle glaucoma (certain cases of Axenfeld–Rieger and Peters Anomaly), and the external approach is not feasible if the state of the sclera and conjunctiva does not allow their manipulation [18].

**Figure 3:**
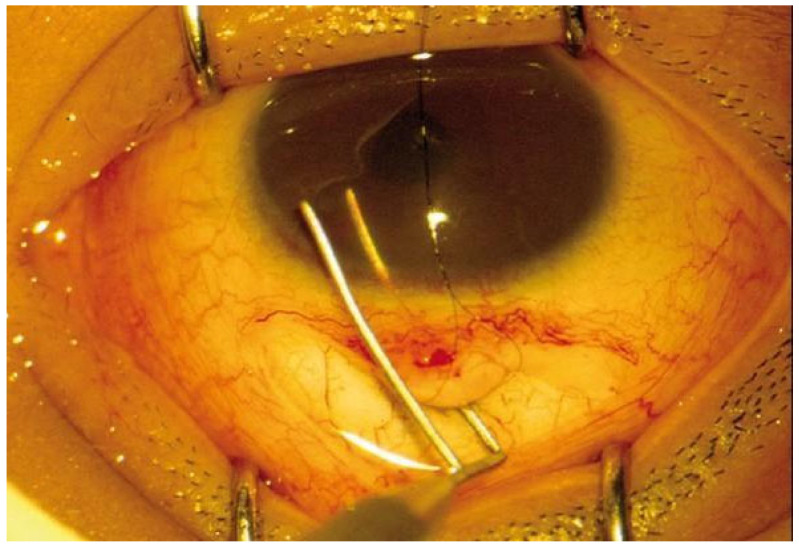
Trabeculotome insertion into the anterior chamber and cutting the walls of the Schlemm’s canal (personal archive of author V.C.).

Similar to goniotomy, the preoperative preparation involves pharmacological miosis with topical pilocarpine [18]. The conventional ab externo trabeculotomy involves a sequence of forming a conjunctival flap and the formation of a partial thickness scleral flap, which is advanced into the corneoscleral junction [18]. Then, deeper dissection is performed until the Schlemm’s canal is identified, and a metal instrument called a trabeculotome is advanced into the anterior chamber, thus cutting the walls of the Schlemm’s canal and trabecular meshwork and easing aqueous humor outflow, Figure 3 [18]. Then, the scleral and conjunctival flaps are sutured watertight [18].

A valuable variation is the ab interno trabeculotomy, also known as Gonioscopy-assisted transluminal trabeculotomy (GATT), performed under goniolens visualization of the iridotrabecular angle. First, a goniotomy knife is used to incise two clock hours of the angle [29]. Then, a 4-0, 5-0, or 6-0 prolene or nylon suture (or, as detailed below, a microcatheter) is inserted into the Schlemm’s canal and advanced until it reaches the other end of the incision (it passes through the entire 360° circumference of the canal). Finally, the two ends of the suture are pulled through the incision and, through traction, perform a 360° trabeculotomy [29].

A more recent development is the introduction of microcatheters, illuminated at one end. These have the significant advantage of easily following the advancing probe and avoiding misdirection into the suprachoroidal space. Moreover, as certain microcatheters have a larger diameter and internal lumen, they allow the insertion of viscoelastic material and viscocanaloplasty [29].

In terms of efficacy, for primary congenital glaucoma and juvenile open-angle glaucoma, an average IOP reduction of 12.5 mmHg was obtained in a case series [30] and a reduction of 44.3–47.9% in JOAG patients [31,32]. Complications may include hyphema, trauma, or perforation of the Descemet membrane, iris root, or ciliary body [18]. The surgical approach to the sclera, as it may be thin and fragile in a buphthalmic eye, may result in leaks, incontinent sutures, or the formation of a filtering bleb [18].

While not as used in pediatric glaucomas as in adult cases, trabeculectomy is another important surgical technique in this population and is also more familiar to ophthalmic surgeons [33]. In primary congenital glaucoma, this technique is indicated as second-line treatment if angle surgery does not achieve IOP control or as first-line treatment if the case is considered a poor candidate for angle surgery or if very low IOPs are required for optical case evolution [18]. This surgical technique represents the first line of treatment in JOAG and in certain secondary glaucomas [18].

### 4.3. Glaucoma Drainage Devices

Glaucoma drainage devices (GDDs) represent valuable therapeutic alternatives for childhood glaucoma patients. These devices are generally composed of a tube into which aqueous humor drains and a plate placed in the subconjunctival space toward which the draining is performed. These components are made of biocompatible acrylic or silicone, and the mechanism can be non-valved or valved, the latter with the advantage of lower risk of postoperative hypotony [18]. Different designs include the Molteno Glaucoma Drainage Device, the Baerveldt Glaucoma implant, and Ahmed Glaucoma Valve implants; more recently, the Aurolab aqueous drainage implant has been introduced as a low-cost alternative [18,27].

GDDs are indicated as a second-line therapy, after angle surgery, or first-line if angle surgery is unlikely to be successful. In the second line, the choice is between GDD and classical trabeculectomy [18]. In terms of surgical techniques, both trabeculectomy and GDDs can be performed in the case of corneal edema and both need surgical intervention at the level of the conjunctiva. While GDDs may lead to a higher average IOP and need supplemental topical treatment, compared to trabeculectomy, the latter is more susceptible to stop working in case other ocular surgeries are performed [18]. In the long term, while trabeculectomy carries a lifelong risk of endophthalmitis, GDDs incur the risk of tube-related complications [18].

Different from the before-mentioned surgeries, GDD implantations require several considerations: axial length may dictate the choice of implant size, conjunctival mobility may dictate the quadrant where the GDD is implanted, and the aspect of the anterior chamber and iridotrabecular angle may dictate the placement of the tube [18].

**Figure 4:**
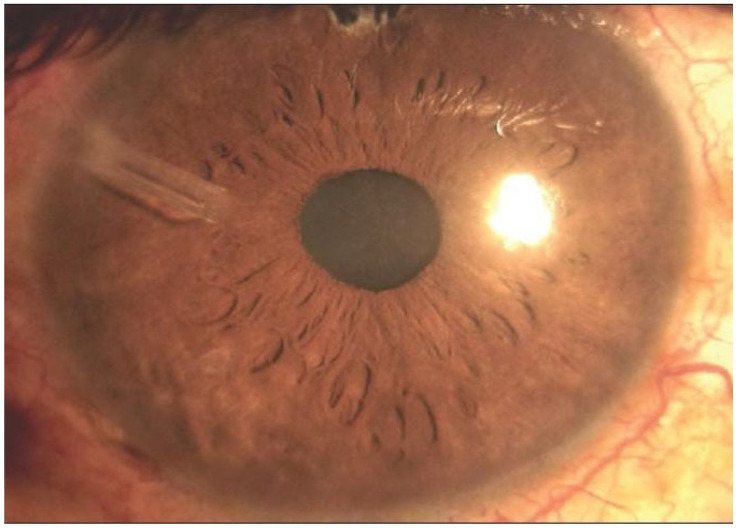
Final aspect of GDD implantation, with a tube placed in the anterior chamber (personal archive of author V.C.).

The surgical technique involves a drop of vasoconstricting adrenaline, a conjunctival incision is performed, the Tenon capsule is dissected and elevated, and the rectus muscle is identified [34]. Then, the device is primed with sterile saline solution and inserted into the conjunctival pocket created and sutured to the sclera [18]. A paracentesis is created into the anterior chamber and the tube is inserted in an oblique manner, and the overlaying conjunctival incisions are sutured, with the potential addition of a graft to ensure device coverage (pericardium, dura, fascia lata, sclera, or cornea) [34], Figure 4.

In patients with PCG, the average IOP decreases with approximately half after Ahmed valve implantation. It is reported that the cumulative probability of success is 97% in the first year of follow-up, decreasing to 56% in the fifth year. Complications may include contact between the tube and the endothelium, development of cataract, tube, or valve expulsion, and retinal detachment [26]. It is reported that, across different GDDs, the success rate is around 80% after 1–2 years of follow–up, with values around 50% in patients followed for longer [35].

### 4.4. Cyclodestructive Procedures

Cyclodestructive procedures are only indicated in advanced cases, with a history of multiple failed surgeries and with a low visual potential [27]. This method is based on the principle of decreasing the aqueous humor production at the level of the ciliary body, as energy absorption in the ciliary epithelium leads to tissue necrosis [36]. It may be applied both trans-sclerally and endoscopically [36]. As the identification of the ciliary processes is more difficult in buphthalmic eyes and as pediatric eyes tend to regenerate the destroyed ciliary processes, several modifications have been developed for these techniques: endoscopic cycloablation and transscleral cycloablation with transillumination to aid the identification of the target structures [27]. Transscleral cyclophotocoagulation (TSCPC), with a diode laser device, was developed in recent years as a safer and more effective method of decreasing IOP. While adverse effects such as phthisis are less frequent, this technique may still induce a certain degree of inflammation [37]. A micropulse approach of this laser allows for a therapeutic amount of energy to be delivered to the ciliary body, and the time between pulses disperses the heat and reduces the risk of inflammation and hypotony [37]. Secondary effects of cyclodestruction may be both undesirable and beneficial to the case evolution: collateral damage to the neighboring trabecular meshwork may impede AH outflow; however, damage to corneal nerve endings may aid the painful symptomatology of certain patients with high uncontrolled IOP [36]. In terms of efficacy, it has been proven that pediatric glaucoma cases often need retreatment of cycloablation: after one treatment with a Continuous Wave Diode Laser, 37% of cases reach an IOP under 22 mmHg or a 30% decrease from preoperative IOP, one year after the procedure. After the second cycloablative procedure, 72% of cases reach this IOP target, one year after the intervention [38]. In the case of MicroPulse Transscleral Laser Treatment, it is reported that 71% of cases had a favorable evolution at 6 months post-procedure, defined as IOP between 5 and 21 mmHg with no vision-threatening complications [39].

Childhood glaucoma is a growing domain in ophthalmology, with ongoing research in this potentially devastating spectrum of disease. Surely, the future of this disease will involve significant innovation, both by improving diagnosis testing and by refining surgical techniques. The difficult collaboration of young patients may be improved with OCT devices, which are handheld or attached to a flexible arm [40]. The genetic landscape of primary glaucoma also needs more research, as currently, tests have only a 40% chance of identifying a genetic cause in a PCG case [41].

Experimental improvements in surgery are also tried: Baerveldt GDD, being extended with a Xen gel stent, acts as an entrance into the anterior chamber and reduces the risk of corneal injury due to tube contact [42]. Another surgical aspect that presents a potential for future research is the high disparity between ethnic populations in terms of angle surgery success, reported as over 80% in Caucasians and under 50% in Asian populations [42]. Similarly, the next line of treatment, which could be GDDs, reports a falling success rate as follow-up continues years after the surgery [42]. This is of particular concern as pediatric glaucoma patients have a long life expectancy and particular visual needs.

## 5. Conclusions

Overall, with prompt diagnosis and adequate treatment and follow-up, the prognosis of PCG may be quite favorable; stationary disease has been reported in 90.3% of cases after one year, 70.8% after 10 years of follow-up, and 58.3% at 34 years [43]. The age of presentation remains a valuable risk factor as angle procedures were 90% successful in cases presenting between 2 and 12 months of age while only 50% successful in late-onset or late-recognized cases [44]. In well-controlled cases, a study reports a median visual acuity in the better eye of 20/30, with a worse visual prognosis in glaucoma following congenital cataract surgery [45].

Immediate recognition of the specific signs and symptoms by caregivers, primary care providers, and ophthalmologists, followed by prompt diagnosis and comprehensive surgical planning, involving the caregivers in the entire process, remains critical for optimizing outcomes in childhood glaucoma management. Congenital glaucoma is a complex disease, and it usually involves follow-up throughout the patient’s life, which may include reinterventions and chronic topical medication. Close collaboration is essential between the child’s caregivers and support circle and the surgical and medical management team.

## Figures and Tables

**Table 1:** Overview of surgical treatment in childhood glaucoma [18].

	Indications	Contraindications	Complications
Angle surgery—Goniotomy	Primary congenital glaucoma Certain cases of Glaucoma associated with non-acquired ocular anomalies, with non-acquired systemic disease, with acquired conditions, following cataract surgery [18]	Insufficient view of the angle (corneal edema) [18]	Hyphema, rarely peripheral anterior synechiae, iridocyclodialysis, lens injury, retinal detachment [25]
Angle surgery—Trabeculo- tomy	Primary congenital glaucoma Juvenile open-angle glaucoma, glaucoma following cataract extraction, certain cases associated with Sturge–Weber Syndrome, glaucoma associated with ocular acquired conditions [18]	Insufficient view of the angle (Ab interno approach) Poor quality of conjunctiva and sclera which endanger the sutures [18]	Ab interno: Hyphema, Descemet membrane, iris root or ciliary body trauma. Ab externo: leaking sutures, filtering bleb [18].
Glaucoma drainage devices (GDD)	As second line after angle surgery if IOP is not controlled, or if angle surgery is not considered feasible [18] At this point in the decision making, the surgeon has a choice between GDD and trabeculectomy with antimetabolites		Tube-endothelium contact, cataract development, tube or valve expulsion, retinal detachment [26]
Cyclodes—tructive procedures	Uncontrolled glaucoma cases, in which it is considered that previous surgical lines are not feasible [18]	Uveitic eyes, scleral thinning (risk for scleral staphyloma), pigmented sclera (risk of conjunctival and scleral burns) [18]	Ocular hypotony, lens opacification and cataract formation, choroidal and retinal detachment [18]

## Data Availability

Not applicable.

## Abbreviations

The following abbreviations are used in this manuscript:

AL	Axial length
BSS	Balanced salt solution
CGRN	Childhood Glaucoma Research Network
GATT	Gonioscopy-assisted transluminal trabeculotomy
GDD	Glaucoma drainage devices
IOP	Intraocular pressure
JOAG	Juvenile open-angle glaucoma
OCT	Optical coherence tomography
PCG	Primary congenital glaucoma
